# Overview of ICARUS—A Curated, Open Access, Online Repository for Atmospheric Simulation Chamber Data

**DOI:** 10.1021/acsearthspacechem.3c00043

**Published:** 2023-05-16

**Authors:** Tran B. Nguyen, Kelvin H. Bates, Reina S. Buenconsejo, Sophia M. Charan, Eric E. Cavanna, David R. Cocker, Douglas A. Day, Marla P. DeVault, Neil M. Donahue, Zachary Finewax, Luke F. Habib, Anne V. Handschy, Lea Hildebrandt Ruiz, Chung-Yi S. Hou, Jose L. Jimenez, Taekyu Joo, Alexandra L. Klodt, Weimeng Kong, Chen Le, Catherine G. Masoud, Matthew S. Mayernik, Nga L. Ng, Eric J. Nienhouse, Sergey A. Nizkorodov, John J. Orlando, Jeroen J. Post, Patrick O. Sturm, Bridget L. Thrasher, Geoffrey S. Tyndall, John H. Seinfeld, Steven J. Worley, Xuan Zhang, Paul J. Ziemann

**Affiliations:** † Department of Environmental Toxicology, University of California Davis, Davis, California 95616, United States; ‡ Center for the Environment, Harvard University, Cambridge, Massachusetts 02138, United States; § Division of Chemistry and Chemical Engineering, California Institute of Technology, Pasadena, California 91125, United States; ∥ Information and Educational Technology, University of California Davis, Davis, California 95616, United States; ⊥ Department Chemical and Environmental Engineering, University of California Riverside, Riverside, California 92507, United States; # Cooperative Institute for Research in Environmental Sciences, University of Colorado Boulder, Boulder, Colorado 80309, United States; ∇ Department of Chemistry, University of Colorado Boulder, Boulder, Colorado 80309, United States; ○ Department of Chemistry, Carnegie Mellon University, Pittsburgh, Pennsylvania 15213, United States; ◆ Department of Chemical Engineering, Carnegie Mellon University, Pittsburgh, Pennsylvania 15213, United States; ¶ Department of Engineering and Public Policy, Carnegie Mellon University, Pittsburgh, Pennsylvania 15213, United States; & McKetta Department of Chemical Engineering, The University of Texas at Austin, Austin, Texas 78712, United States; †† Data Stewardship Engineering Team, National Center for Atmospheric Research, Boulder, Colorado 80307, United States; ‡‡ School of Earth and Atmospheric Sciences, Georgia Institute of Technology, Atlanta, Georgia 30332, United States; §§ Department of Chemistry, University of California Irvine, Irvine, California 92697, United States; ∥∥ School of Chemical and Biomolecular Engineering, Georgia Institute of Technology, Atlanta, Georgia 30332, United States; ⊥⊥ School of Civil and Environmental Engineering, Georgia Institute of Technology, Atlanta, Georgia 30332, United States; ## Atmospheric Chemistry Observations and Modeling, National Center for Atmospheric Research, Boulder, Colorado 80305, United States; ∇∇ Air Quality Research Center, University of California Davis, Davis, California 95616, United States; ○○ Division of Engineering and Applied Science, Calif. Institute of Technology, Pasadena, California 91125, United States

**Keywords:** atmospheric chamber, database, data repository, data science, atmospheric chemistry and physics

## Abstract

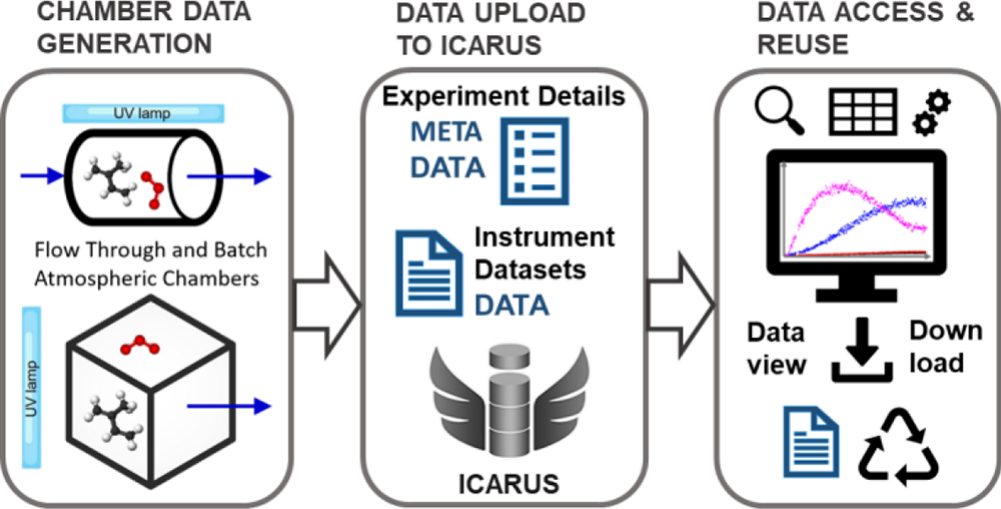

Atmospheric simulation chambers continue to be indispensable tools for research in the atmospheric sciences. Insights from chamber studies are integrated into atmospheric chemical transport models, which are used for science-informed policy decisions. However, a centralized data management and access infrastructure for their scientific products had not been available in the United States and many parts of the world. ICARUS (Integrated Chamber Atmospheric data Repository for Unified Science) is an open access, searchable, web-based infrastructure for storing, sharing, discovering, and utilizing atmospheric chamber data [https://icarus.ucdavis.edu]. ICARUS has two parts: a data intake portal and a search and discovery portal. Data in ICARUS are curated, uniform, interactive, indexed on popular search engines, mirrored by other repositories, version-tracked, vocabulary-controlled, and citable. ICARUS hosts both legacy data and new data in compliance with open access data mandates. Targeted data discovery is available based on key experimental parameters, including organic reactants and mixtures that are managed using the PubChem chemical database, oxidant information, nitrogen oxide (NOx) content, alkylperoxy radical (RO_2_) fate, seed particle information, environmental conditions, and reaction categories. A discipline-specific repository such as ICARUS with high amounts of metadata works to support the evaluation and revision of atmospheric model mechanisms, intercomparison of data and models, and the development of new model frameworks that can have more predictive power in the current and future atmosphere. The open accessibility and interactive nature of ICARUS data may also be useful for teaching, data mining, and training machine learning models.

## 1 Introduction

**Figure 1:**
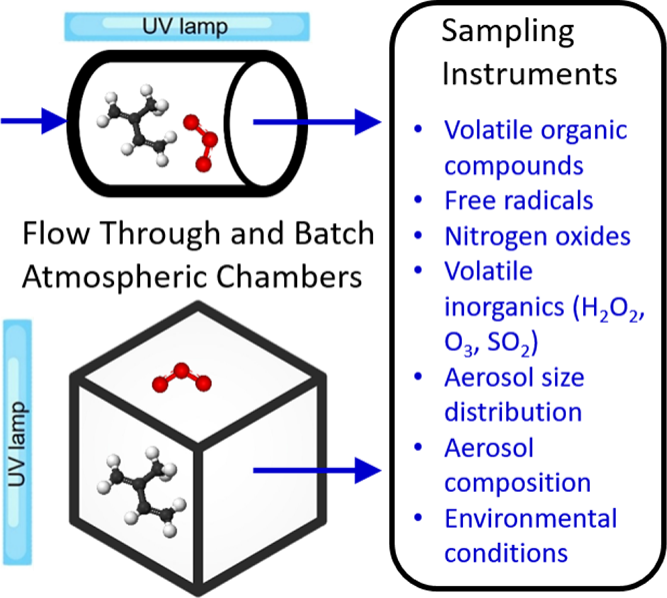
Simplified schematic of atmospheric simulation chambers and their measurements.

Atmospheric simulation chambers (e.g., “smog” chambers, environmental chambers, flow reactors, continuously stirred reactors, etc.; Figure 1) are central to the laboratory study of atmospheric chemistry and physics.^1^ These chambers serve as the critical link between “bench-top” laboratory research and ambient research by enabling scientists to study atmospheric chemistry at relevant time and length scales, but in a highly controlled manner.^2^ These simulation chambers of variable or fixed gaseous volume (ranging from less than 1 m^3^ to more than 200 m^3^) are used to investigate reactions that encompass all phases of atmospheric matter (gaseous, particulate, aqueous, or mixed phases) and test the impacts of temperature, pressure, relative humidity, irradiation with ultraviolet or other wavelengths of light, oxidative exposure, and other factors on chemical reactions. Starting from the pioneering experiments of Haagen-Smit,^3^ atmospheric chamber research has led to important discoveries in atmospheric chemistry, for example, new chemical mechanisms, quantification of rate coefficients and yields, and key insights into reaction dynamics. The fundamental data obtained from chamber studies are routinely used in model mechanisms,^4−8^ which define the known chemistry and empirical constraints in atmospheric chemical transport models that predict the chemical composition of the atmosphere and its health and climate feedbacks. See the Section 2 for a description of a general chamber reaction.

The new U.S. federal open access mandate^9^ and the International Science Council’s mission^10^ to promote the open availability of data for research has amplified the need for open access data repositories in multiple disciplines. There are numerous atmospheric chamber facilities in the world, and more are currently under construction, yet there is no central data management and storage infrastructure for the vast amounts of highly heterogeneous information produced from chamber research within the United States and many parts of the world. Unlike observational field data and model revisions, laboratory chamber data are not routinely made accessible to the research community at large, nor to the public. Such a data infrastructure would also serve as a critical quality control mechanism and enable the facile synthesis of proprietary data across multiple chamber studies for important use applications such as model mechanism development, training machine learning algorithms, or teaching. A data repository and access hub was recently developed for the atmospheric chambers in Europe through the Eurochamp project.^11^ Considerable community interest in developing such a project has been also expressed by scientists in the United States and beyond.^12,13^ Finally, these efforts would shield atmospheric chamber experimental work, which started around the 1950s with the discovery of the reactions in smog,^14^ from being lost when research labs retire.

ICARUS is a collaborative effort from the experimental atmospheric chemistry and physics community to build the first open access, searchable, web-based infrastructure for storing, sharing, searching, and utilizing atmospheric chamber data in North America. The overarching scientific goal of the project is to promote collaboration between atmospheric science researchers and facilitate the sharing and reuse of publicly funded data by increasing uniformity and expanding access to atmospheric chamber experiments. Furthermore, an important fundamental goal of ICARUS is to support the data principles of FAIR (findable, accessible, interoperable, and reusable).^15,16^ The pilot cohort of the ICARUS project included approximately 30 chamber and flow reactor experimentalists from the California Institute of Technology, Carnegie Mellon University, Georgia Institute of Technology, Harvard University, National Center for Atmospheric Research (NCAR), the University of California (UC) at Davis, Irvine, and Riverside, the University of Colorado at Boulder, and the University of Texas at Austin. The Scientific Steering Committee (SSC) of ICARUS is composed of the principal investigators of each research group; the SSC provided feedback and formal votes on all decisions. The repository web infrastructure was developed by the research group at UC Davis, working closely with the Data Stewardship and Engineering Team at NCAR. Each participant group in the pilot voted on project decisions and collaboratively tested each alpha version of the repository until a stable beta version emerged. The beta version was then tested by model mechanism developers (see Section 4). Comments and suggestions by all testers were implemented into the current version of the ICARUS website.

This paper provides an overview of the ICARUS data repository, including the goals of the project, functions, and features of the web interface, organizational system, data infrastructure and formats, technical considerations, and anticipated uses.ICARUS contains two interactive parts: a Data Intake portal and a Data Access portal. In this overview, we term the Data Contributor as a person who uses the Data Intake portal to provide manual entries of metadata descriptors and to upload data files on behalf of a group or organization. The Data User is defined as any person who uses the Data Access portal to view and/or download the data for reuse for any purpose. A key insight by the Eurochamp project is that ample metadata describing each chamber experiment is critically needed for Data Users to accurately interpret the data and lower the barrier to reuse.^17^ ICARUS has focused on making these extensive metadata available for Data Users, while at the same time decreasing unnecessary work for the Data Contributor through automation of repetitive tasks where feasible.

## 2 Experimental Section

This section describes one example of a typical chamber experiment that may be hosted on ICARUS. Generally, atmospheric chambers are used to isolate reactions that would otherwise occur concurrently in the ambient atmosphere for a detailed study. Experimental methods that generate atmospheric chamber data will vary with the type of chamber, type of gaseous mixing mode (plug flow, batch, or continuously stirred reactors), experiment goals, type of reactant, and type of analytical instruments. Detailed overviews of atmospheric simulation chamber experimental methods are reported elsewhere.^18−25^ A typical application of a cubical Teflon chamber (Figure 1, bottom) equipped with ultraviolet lights run in batch (stopped flow) mode is described briefly here. Consider, for example, a reaction between the volatile organic compound isoprene (C_5_H_8_), with the hydroxyl radical (OH) under a “non-polluted” chemical regime, where the organic peroxy (RO_2_) radicals react primarily with hydroperoxy (HO_2_) radicals.^26^ Chemical reagents such as hydrogen peroxide (H_2_O_2_) and isoprene are injected at the start of the reaction at the desired concentrations. Concentrations of volatile compounds in the chamber are generally monitored as a function of reaction time by in situ chemical instruments such as gas chromatography-flame ionization detectors or chemical ionization mass spectrometers. When measuring secondary aerosol yields, seed particles (such as ammonium sulfate) are typically atomized into the chamber to serve as the surfaces to which condensable vapors may partition. Particle concentrations and/or composition are monitored by in situ particle sizers and counters, aerosol mass spectrometers, particle into liquid samplers, or other analytical techniques. Either ultraviolet or simulated solar lights are turned on to initiate radical formation, e.g., photolysis of H_2_O_2_ and subsequent reactions will produce both OH and HO_2_ radicals. The decay of reactants and generation of products are monitored for the duration of the experiment, often together with environmental conditions (temperature, relative humidity, and pressure). Lights are turned off after the objectives of the experiments are met, and then gases and particles might be sampled for offline analyses. Dark reactions (using ozone, nitrate radical, or other dark oxidants) may also be tested in atmospheric chambers in the absence of irradiation. The data from chemical and particle instruments are then processed for later use and may be uploaded onto ICARUS together with experimental details.

## 3 Results: The ICARUS Framework

This section describes the user interfaces and technical process for the intake and access of data and metadata through the ICARUS website.

### 3.1 Data Intake

Data intake is the process by which the Data Contributor categorizes and describes their scientific datasets and uploads the associated data. The data intake process is access-controlled with login access from user accounts. Please see the ICARUS user guide (Section S1) for technical details about the data intake process.

#### 3.1.1 Technical Objectives

One of the primary technical goals of ICARUS is to adopt a data format standard that unifies the various file types and structures that come from the many analytical instruments available for atmospheric research (e.g., .xlsx, .txt, .hdf, .mat, .itx, and .ict). This enables Data Users to treat all downloaded data similarly, with the same software and reader script. Similarly, ICARUS seeks to digitize experimental metadata and adopt a uniform standard for metadata reporting from different research labs. This information may include descriptions of chambers, experiment goals, instrument sampling protocols, analytical uncertainties, and other information necessary for the Data User to accurately interpret the experiment data. In that spirit, the metadata requirements of ICARUS are high. Users are required to extensively describe each measurement, experiment, and any linked entities; however, the process for data entry and uploads is streamlined to minimize repetitive work for the Data Contributor. Machine-based quality assurance checks are built into the data entry and upload process (e.g., minimum word requirements for descriptions of experiments, data file column checks, and required fields). Archiving legacy data with high amounts of metadata is a first priority, after which focus turns to managing current and future datasets. Given the increase in FAIR-aligned data availability requirements of large scientific publishers during the data submission or publication stage,^27−31^ ICARUS is best used by the Data Contributor as an integrated part of the data workflow from experimentation to publication.

#### 3.1.2 Data Ecosystem

**Figure 2:**
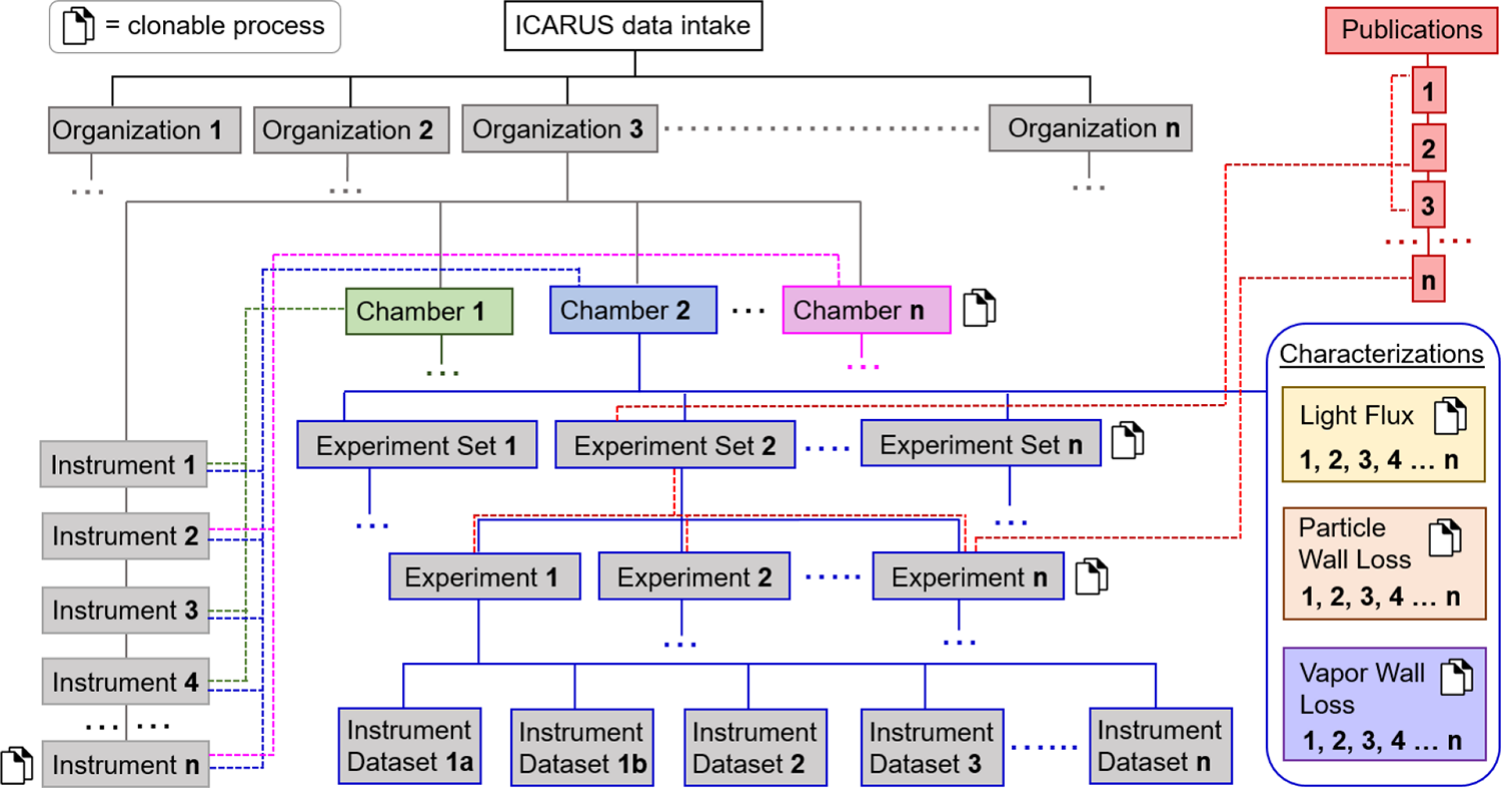
Schematic of how each entity relates to another in the ICARUS data ecosystem. Enumerations start from 1 and end in *n* (any number) for each entity. Publications are shown linked only to Experiment Set 2 for brevity, and Characterizations show no linkages; however, both can be linked to any Experiment Set or Experiment. Characterizations are also linked to Instruments because they require a dataset upload.

The ICARUS data ecosystem includes a set of entities that are related to one another via parallel and hierarchical relationships, all of which are internally tracked (Figure 2). Data Contributor-provided descriptions of these entities are stored as the repository metadata and may be updated at any time.

At the top of the hierarchy is the Organization, which describes the research group, organized research unit, or other organizational entities to which the Data Contributor belongs. Immediate descendants of the Organization are the Instruments and Chambers. Instruments describe the analytical sensors and other scientific equipment that record the data uploaded to ICARUS. These Instruments may provide either in situ or “offline” measurements that characterize the chemical reactions or physical phenomena that are tested in the atmospheric chambers (e.g., concentrations of chemicals or particles, composition of chemicals or particles, spectral irradiance or absorbance, temperature, relative humidity). Chambers are at the same hierarchical level as Instruments. Chambers describe physical characteristics and other properties of the atmospheric chambers or flow reactors (as well as surrounding infrastructure), in which the scientific experiments are performed. These characteristics of the Chamber may include size, chamber material, environmental controls, a description of the lamps, and so forth. Instruments may be linked to multiple Chambers within ICARUS; a linkage will define the analytical datasets that will be available to upload for each experiment performed in that Chamber. The immediate descendants of Chambers are Experiment Sets, which are groupings of individual experiments based on logic defined by the Data Contributor (Section S1.D).

The immediate descendants of Experiment Sets are the Experiments, including any background/control experiments, which are the records of the individual scientific investigations within the atmospheric chamber. The Experiment level has the most information available for Discovery purposes (Section 3.2) and, thus, more descriptions and supplemental information are provided at this level (Section S1.E). Besides descriptions of the Experiment categories, goals and outcomes, input of reactants, and other information about the Experiment (collectively termed the Experimental Metadata), there is a requirement for the upload of comma-separated value (csv) files: the Instrumental Dataset file(s) and Timeline file. The Instrumental Dataset files are a record of the data that have been processed to their final ready-to-use forms (e.g., raw signals that have been baseline-corrected, calibrated, and converted to concentration units). The Timeline is a two-column chronological list of experimental actions that occurred during the Experiment; it is provided so that the data can be appropriately interpreted by the Data User. For example, increases or decreases in the signal of certain compounds may be associated with the initiation of chemistry by turning on lights; similarly, discontinuities in data may be associated with instrument sampling issues.

In our experience, digitizing the experimental Timeline is a significant hurdle to the archiving of legacy atmospheric chamber data. The majority of experiment notes that exist in the community have been recorded in physical notebooks, often by a large rotating roster of academic or government personnel. Thus, the quality, quantity, and availability of such notes vary between labs and between research experimentalists within the labs. A more sustainable data management practice includes planning for the ICARUS upload in the data workflow by recording notes digitally during the experiment by using electronic laboratory notebooks or similar tools,^32,33^ by using the Timeline generator tool or template available from ICARUS (Section 3.2.3), or by digitizing notes soon after an experiment.

##### 3.1.2.1 Controlled Vocabulary

We control the vocabulary of many metadata fields through various means: for example, calendar entries for dates, radio buttons for yes and no, drop down menus with limited selections, and check boxes for multiple input terms. Table 1 provides the available terms for the non-freeform fields, where the vocabulary is controlled. These terms provide suitable keywords for sorting and searching.

**Table 1:** Experimental Fields and the Associated Controlled Vocabulary^a^

Experiment category(s)	Gas phase chemical reaction, condensed phase chemical reaction, multiphase chemical reaction, volatility and partitioning, hygroscopicity and phase changes, aerosol formation, aerosol aging, instrument/chamber characterization, blank/control, custom
Reaction type(s)	Non-chemical, photooxidation, hydrolysis, oligomerization, heterogeneous oxidation, other aqueous/aerosol phase, custom
Reactant name(s)^#^	Any substance or compound from PubChem^#^, custom (free form entry)
Reactant functional group(s)	Aerosols, alcohol, aldehyde, alkane, alkene, alkyne, amine, anhydride, aromatic, cycloalkane, cycloalkene, diol, ester, ether, halocarbon, hydroperoxide, imine, inorganic acid, ketone, N-heterocycle, NOx, nitrate, nitrile, nitroalkane, nitroaromatic, O-heterocycle, organic acid, peracid, peroxyacid, sulfate, sulfide, sulfoxide, others
Seed	Ammonium sulfate, ammonium nitrate, sodium sulfate, sodium chloride, SOA, N/A, custom
Oxidant	None, hydroxyl radical, ozone, nitrate radical, chlorine radical, O atom, custom
RO_2_ main fate^#^	HO_2_, NO, RO_2_, not sure, custom

a Field names marked with a ^#^ are described in more detail in the text.

Chemical reactant names and their synonyms in ICARUS are managed by the suite of controlled vocabulary from the PubChem databases,^34^ the world’s largest collection of freely accessible chemical information. It is necessary to manage chemical vocabulary because numerous synonyms exist for each chemical compound. The chemical synonyms result from variations in spelling or notation (e.g., HCHO, CH_2_O, formaldehyde, and methanal), chemical site designations (e.g., isopropanol vs 2-propanol), and prefix symbols (e.g., b-myrcene, ß-myrcene, and beta-myrcene). This poses significant challenges for the search and discovery process, as incomplete search results will appear when Data Users query ICARUS using only one of the many existing synonyms. The usage of PubChem in ICARUS is beneficial because this allows the user to enter any known synonym for a certain compound/mixture into the data entry field, instead of needing to look up controlled names defined by other existing standards, for example, IUPAC or CAS. The user-input chemical entries will then be automatically validated by ICARUS by a rapid query with the PubChem Compound and Substance databases and updated onscreen with the “preferred” or “common” name in PubChem for that chemical or substance.

##### 3.1.2.2 RO_2_ Main Fate

In support of model mechanism development, a mechanism-focused sorting field called “RO_2_ main fate” is used. Alkylperoxy radicals (RO_2_) are formed in an oxidation reaction of hydrocarbons initiated by the OH, Cl, O, and NO_3_ radical or by ozonolysis to various extents. Their “fate,” or predominant bimolecular or unimolecular reaction, determines the chemical regime and thus product formation for that hydrocarbon precursor.^35,36^ The “RO_2_ main fate” describes whether most of the first-generation RO_2_ radicals of that oxidation reaction react with NO and HO_2_ radicals, NO_2_ radicals, and other RO_2_ radicals or via autoxidation (isomerization).^37,38^ Custom entries are provided for experiments that study a different and/or less common fate, for example, with OH radicals or NO_3_ radicals. This criterion replaces the more traditional “NOx regime” used in atmospheric chemistry, which has been noted as ambiguous.^39^ The “General RO_2_ Fate Estimator” program (Section 3.2.3) available on the ICARUS website uses IUPAC-recommended rates^40^ to estimate RO_2_ regimes based on user inputs of oxidant and hydrocarbon loadings.

Finally, each Experiment or Experiment Set can be linked to Publications and Characterizations in ICARUS. A Publication is a metadata record of published works in the scientific literature that are associated with the experimental data that were uploaded by the Data Contributor. Characterizations are experiments that are performed for the purpose of quantifying chamber wall loss coefficients of vapors and particles or the emission fluxes of chamber lights under various environmental conditions of the main Experiment. Chamber characterization data are often required for modeling the photochemistry and product formation in chamber experiments, and thus, are critical supplemental data for each Experiment or Experiment Set.

#### 3.1.3 Task Automation and Saving

ICARUS is designed to offer both simplicity for the Data Contributor and comprehensiveness for the Data User. These goals are achieved by requiring extensive dataset descriptions and metadata as described in Section 3.1.2, while automating many repetitive tasks and saving progress to minimize lost work.

In order to increase efficiency in data entry and uploads, ICARUS provides a “Cloning” feature for duplicating Chambers, Instruments, Experiment Sets, Experiments, and Characterizations if the user seeks to make few changes to the entity (Figure 2, cloning icons). For example, a series of chamber experiments may seek to perform the same reaction but with different temperatures, relative humidities, seed particle surface area, or any other variable experimental parameter. In these cases, the Data Contributor will clone an experiment and change the field value only for the relevant parameter that was altered in the experiment. Experiments may also be moved to different Experiment Sets to allow for reorganization with the “Move” feature. Experiment names are automatically generated with the same syntax (which includes the Organization name, Experiment date, and other information) to save time and to increase uniformity. Data Contributors may also save their progress and return to data entry due to the Draft mode in ICARUS, which does not publish the data and ignores any errors from incomplete fields.

#### 3.1.4 Data Format

When an Experiment or a series of Experiments are downloaded, ICARUS writes the data into a ASCII-based file format called YAML (Section S1.I) that is meant to be read into data processing programs written in, for example, R, Matlab, Python, or another scripting language, for further use by Data Users. An example of the data format is shown in Figure S2. A downloaded data packet (compressed .zip file) for one single Experiment contains the Experimental Metadata, all associated Instrument Datasets, all associated Characterization files, and a manifest file that describes the content of the folder and the data location on the ICARUS website. Multiple unrelated Experiments can be downloaded together in a single .zip file organized by Organization name.

The download data format of ICARUS is unique, which better serves the needs of the discipline, but may necessitate new data reader tools to be written. Fortunately, ICARUS can leverage the many free and open source data readers available for YAML, so we do not consider this to be a big disadvantage. Adoption of existing earth science data and metadata format standards for ICARUS proved challenging due to the lack of laboratory-specific, and particularly chamber-specific, fields and keywords. For example, the ICARTT format^41^ from the National Aeronautics and Space Administration (NASA) is widely used for atmospheric field measurements. However, ICARTT caters to continuous measurements of the natural environment instead of experiment-based chamber research performed at intermittent frequency. The Global Change Master Directory (GCMD) includes a number of earth-science related keywords; however, insufficient laboratory-related keywords were available. The ICARUS data format integrates some GCMD keywords with other controlled vocabulary specific to atmospheric chamber experiments and with chemical vocabulary from PubChem.

### 3.2 Search and Discovery

**Figure 3:**
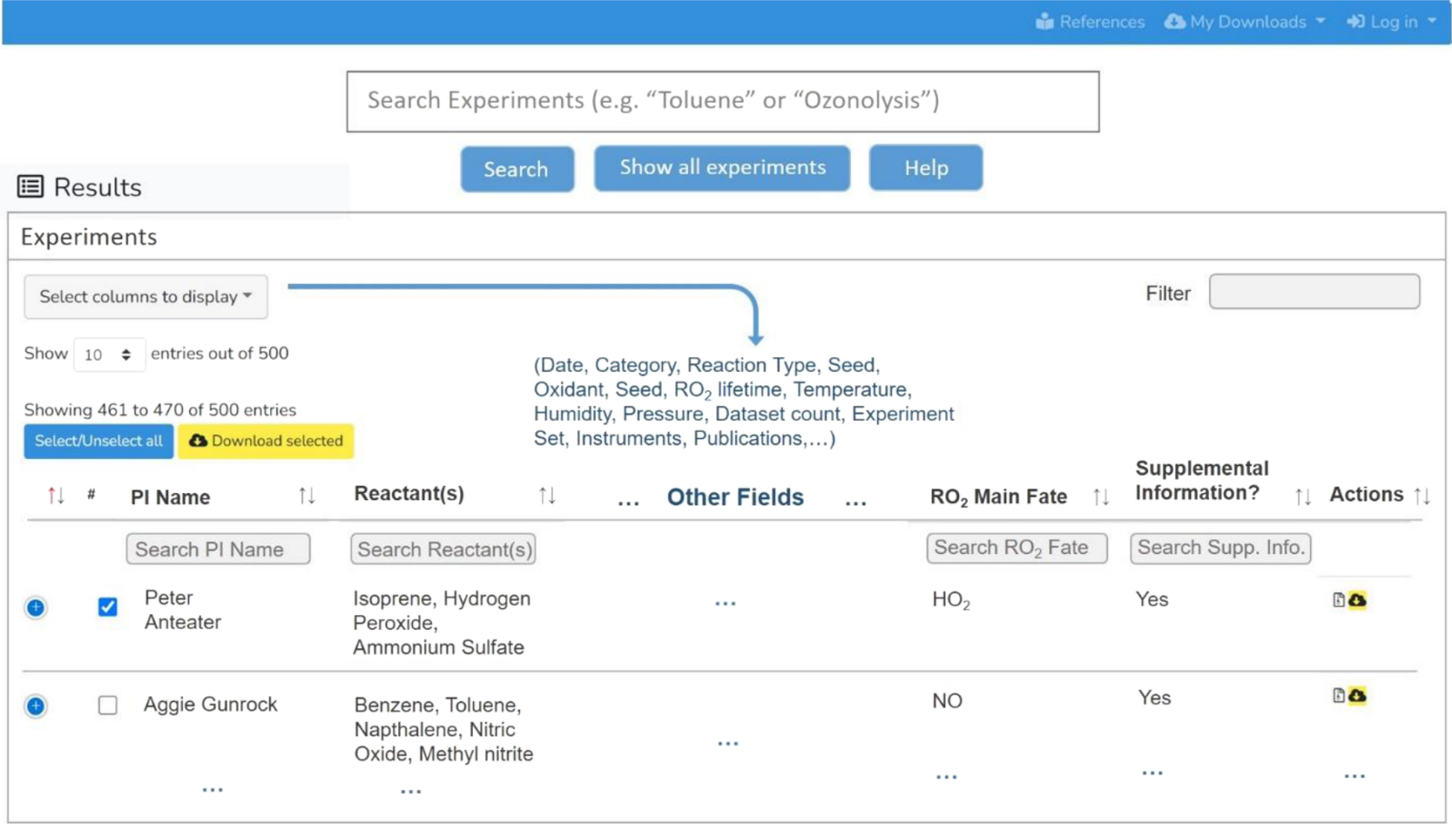
Illustrative search and discovery table from the ICARUS website.

ICARUS is also a search and discovery platform for atmospheric chamber data. The data search, browse, and sort functions in ICARUS are optimized for model mechanism development. Data Users can find data in two ways: a keyword search on the home page and the search results page, as well as browsing all data (“Show All Experiments”) and then utilizing the filter, sorting, and category-specific search functions on the results page (Figure 3).

From the search and discovery table, the Data User can customize their viewing experience and further refine their search. The search and discovery table shows 12 default columns that are categories from which the Data User can narrow down their queries. Each category column is searchable with its own search box and sortable alphabetically. The default columns are chosen to represent categories that would appeal to the majority of users, for example, we expect most Data Users would want to search for keywords within the Experiment Name, PI name, Date, Experimental Category, Reaction Type, Reactant Name(s), Seed Name(s), Oxidant Name(s), RO_2_ Main Fate, Temperature, Humidity, and whether Supplemental Information is available for that Experiment. In total, there are 22 categories that can be selected/deselected to show on the results table by clicking on the “Select columns to display” button, and these user preferences are retained on the website regardless of login status.

It is challenging to view many columns of metadata in a traditional scrolling format; thus, we included an “Expand” button (the blue plus sign, Figure 3) that shows hidden or collapsed metadata as an expanded list in the vertical direction without the need to scroll. This expansion function makes the search and discovery process friendly for mobile devices.

#### 3.2.1 Viewing Experiments

**Figure 4:**
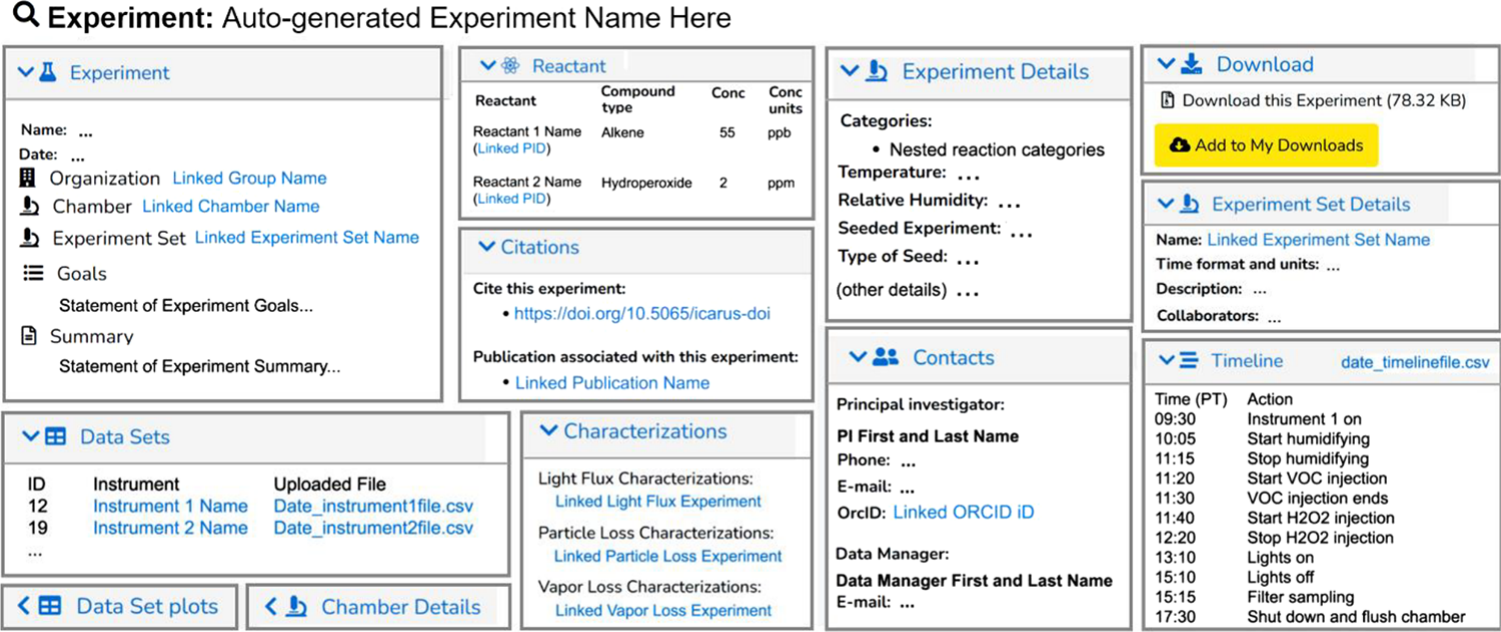
Simplified diagram of the Experiment View webpage from the ICARUS website. Certain noted entities are linked to their respective webpages with persistent url. Each csv file can be downloaded directly by clicking on its name. Most panels are abridged for brevity. Chamber Details and Data Set Plots panels are shown collapsed. The configuration of open/collapsed panels is automatically saved for each Data User. An expanded Data Set Plot panel is shown in Figure 5. A representative example of an Experiment page in ICARUS is shown in Figure S1 for Experiment number 691.

Visitors to the website may download data through multiple mechanisms (Section S1.J) or click on an experiment for additional details before deciding to download the data. This takes them to the Experiment View page (Figures 4 and S1), which shows all of the associated metadata, uploaded Timelines and Dataset, links to parent entities (Organization, Chamber, and Experiment Sets), links to associated Publication and Characterizations, links to Reactant PubChem ID (PID), and a link to download the Experiment, each as separate collapsible panels. The Experiment View page also allows direct downloads of individual Timeline and Dataset .csv files by clicking on their names. As Data Users may have different needs for the information available, the ICARUS website will remember the configuration of collapsed or expanded panels that were set up, without the need for a login, in order to optimize the unique viewing experience of the Data User.

**Figure 5:**
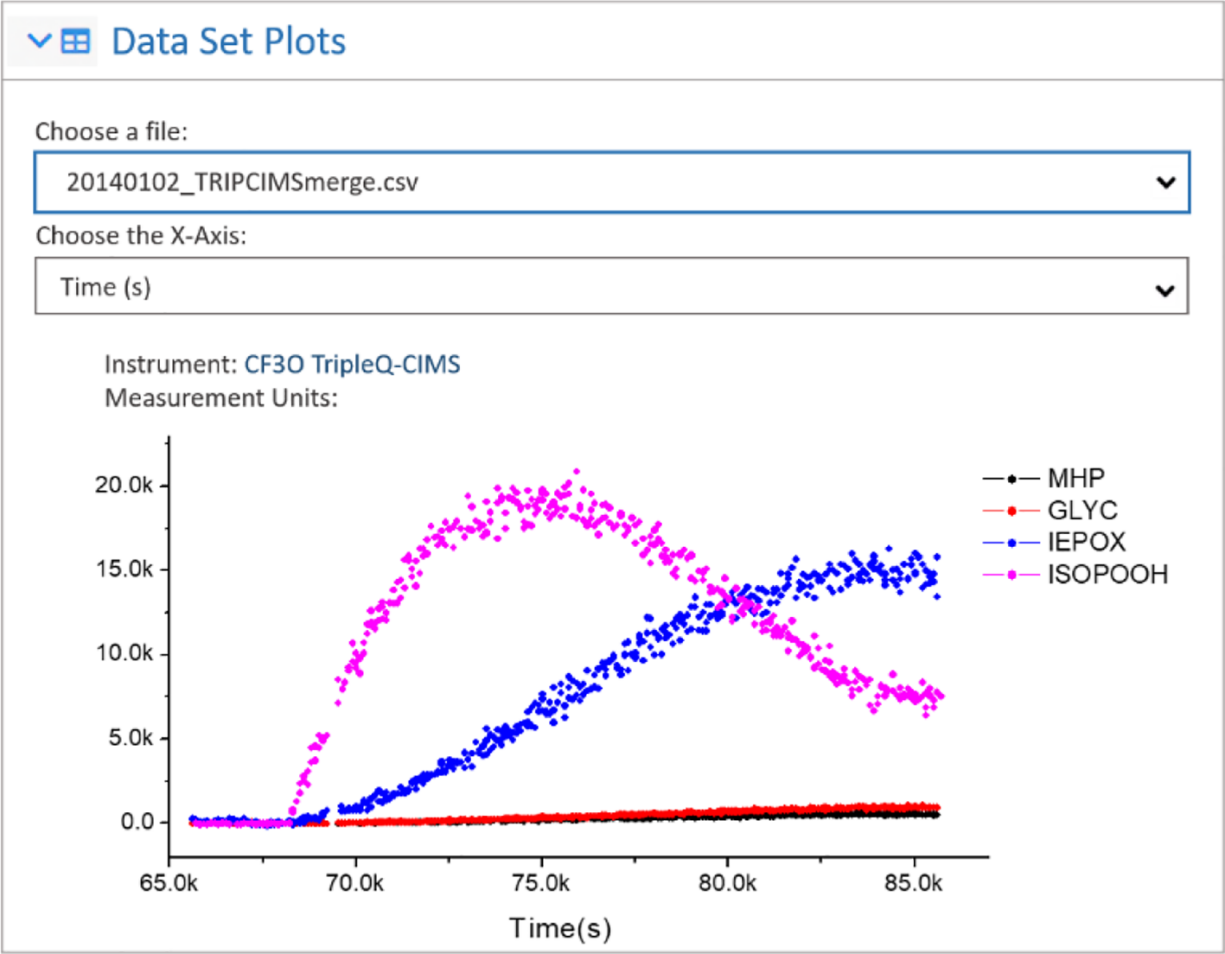
Representative data visualization panel results from the ICARUS website. Available data files are selectable with a drop down menu, and within that data file, all columns are plotted. Users may choose their ideal *x*-axis for plotting and choose data columns to show or hide. Nomenclature and units are defined by Data Contributors in the Instrument metadata for each dataset.

Data Users can also visualize each dataset within an experiment with the interactive Data Plots feature (Figure 5) prior to deciding to download the Experiment. Upon selection of any available dataset, all data columns from that dataset are plotted. Large datasets are automatically down-sampled to display more quickly. Users can select their preferred independent (*x*-) axis from a drop-down list of column names, which is useful in situations where there are multiple potential independent axes. Columns of data can be turned on and off by clicking on the legend, which removes unwanted data on the plotted axis. The data acronyms are defined in the Instrument and/or Experimental metadata.

#### 3.2.2 Shared Tools and Documentation

A number of freeware tools that were contributed by the community are also available to download on the repository website. These tools help Data Contributors with various steps in the data upload workflow. For example, Timeline generator tools, Timeline templates, RO_2_ fate estimator tool,^42^ and Oxidation flow reactor exposure estimator tool^43^ help generate necessary files and metadata information for input into forms throughout the Data Intake process. A 30-min video guide is available to document the process of Data Intake and Access. The video and its transcribed script in English are available through the “Help” button on the front page and the search results page (https://icarus.ucdavis.edu/help). This video describes the entire process from signing up for an account to submitting data. Additional help is available by contacting the ICARUS team.

#### 3.2.3 Mirroring and Indexing

All data in ICARUS are automatically discoverable and version-tracked at the Geoscience Data Exchange repository (GDEX, https://gdex.ucar.edu/search.html?q=icarus) that is operated by the National Center for Atmospheric Research (NCAR). All data from ICARUS experiments are mirrored within GDEX and receive a DOI for persistent identification, location, and citation. ICARUS is synced with GDEX daily. Changes to data files and metadata from ICARUS experiments are tracked within GDEX as separate versions. Prior versions are retained and available upon direct request to GDEX, but are not exposed to data users to prevent confusion. ICARUS data, including the versions mirrored at GDEX, are indexed on Web of Science and Google for discovery through general searches.

## 4 Discussion: Applications for the Reuse of ICARUS Data

Some of the major applications of ICARUS involve modeling, for example, evaluation and revision of current model mechanisms, intercomparison of model mechanisms, and development of new frameworks for modeling atmospheric chemistry. In order to optimize the search and discovery experience and validate data/metadata quality for modeling, ICARUS underwent a technical review and revision process with 13 international model mechanism developers from Harvard University, NCAR Atmospheric Chemistry Observations & Modeling group, Columbia University, UC Riverside, the US Environmental Protection Agency, Colorado State University, the University of Texas at El Paso, NASA Goddard Flight Center, the University of Cambridge, the University of York, and the National Oceanic and Atmospheric Administration (NOAA). The data in ICARUS were tested against the following models: GAMMA,^44^ F0AM,^45^ MusicBox,^46^ SOM-TOMAS,^47^ AtChem with MCM,^48^ WRF-Chem,^49^ GECKO-A,^50^ BoxMox^51^ and various custom models in R, Python, and Matlab. Modelers provided ratings and specific feedback regarding the repository website, including ease of access and navigation, usability of the search and browse functions, quality of data and metadata (including relevancy, completeness, etc.), quantity of metadata (including if there was enough information in the files and names to support the use application), display aesthetics, and multiple other considerations. Feedback from the scientific community of both Data Contributors and Data Users were incorporated into the current version of ICARUS.

ICARUS can also be used by instructors who wish to integrate public data to teaching atmospheric chemistry. Using data to teach may be helpful for classrooms that do not have the infrastructure to conduct these sophisticated experiments, but desire an active-learning model for teaching abstract concepts in atmospheric science such as photochemistry, heterogeneous reactions, kinetics, or surface deposition. Many classical experiments are available, such as VOC-NOx-O_3_ reactions and the photo- and dark oxidations of hydrocarbons. Many of these experimental data are accompanied by published scientific articles that can be used for literature review by students. In particular, the interactive data plotting feature of ICARUS offers a valuable hands-on experience for students that can be compatible with guided discovery models of pedagogy. Based on the experimental conditions and timeline, a student will be able to interact with each data product that had been collected and understand the cause and effects of experimental actions, helping to solidify their learning. Students will be able to download data to practice quantitiative concepts in data analysis, for example, extracting kinetic constants from reactant decay plots or calculating the timescale of particle loss and coagulation.

Regarding the development of new model frameworks in research, one potential research need is a way to parameterize the oxidation chemistry of volatile organic compounds (VOC) in the atmosphere to relevant current and future scenarios. Such scenarios may include (1) increases in temperature due to exacerbation of the climate crisis (with related changes to relative humidity) that impact air pollutant concentrations;^52^ (2) transitions to green energy in cities that amplify the air quality importance of VOC emissions from more diverse sources (e.g., a variety of biogenics, volatile chemical products (VCPs), cooking emissions, fire emissions, and other sources) at the expense of emissions from combustion engines;^53^ and (3) further reductions in NOx emissions that increase the lifetime and change the fate distributions of the RO_2_ radical intermediate in the atmosphere toward autoxidation.^54^

**Figure 6:**
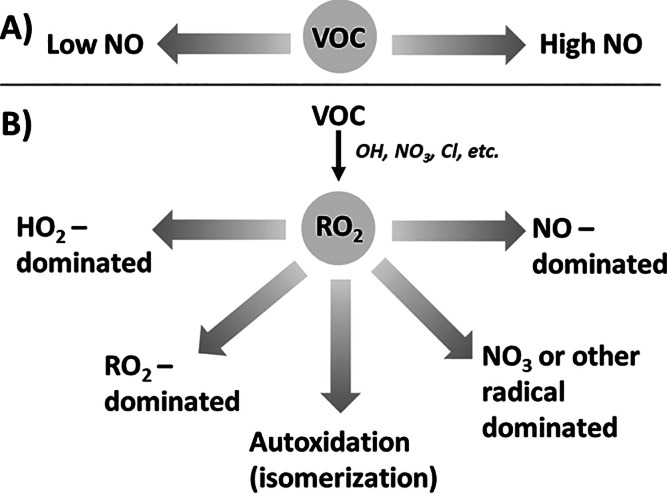
Simplified diagram of model parameterizations of the dependence of atmospheric oxidation chemistry on nitric oxide emissions: (A) Dichotomy of low and high NO regimes; each one may incorporate more than one distinctive reactive pathways for the volatile organic compound (VOC) precursor; and (B) more explicit representation of fates of the alkylperoxy (RO_2_) radical intermediate from VOC oxidation with branching toward more diverse chemical regimes that exist in the current and future atmosphere.

Regarding scenario 1, ICARUS has categorized data explicitly by temperature and relative humidity, which supports community efforts to advance the understanding of atmospheric aerosol and gas-phase processes in a warmer atmosphere. Regarding scenario 2, the integration of the ever-expanding PubChem registry of chemicals and substances in ICARUS supports the reporting of data from new chamber experiments using highly diverse VOCs (such as those in the VCP family of compounds), VOC mixtures (such as fire emissions), and other chemicals of emerging importance. Regarding scenario 3, current model mechanisms do not represent autoxidation well due to NOx-dependent parameterizations [which generally refer to dependences of nitric oxide (NO) instead of the total NOx; Figure 6A]; these were developed because other RO_2_ fates were not yet recognized to be important historically.^55^ The lower availability of data and parameterizations for autoxidation and RO_2_ + RO_2_ reactions have also precluded the widespread inclusion of these processes in simplified SOA models; however, more RO_2_-focused data are becoming available from chamber experiments to support mechanism revisions.^56−59^ It has been shown that understanding the RO_2_ reactivity is important for accurately representing the formation of secondary organic aerosol, organic nitrogen species, and other products in some chemical systems.^60,61^ ICARUS enables the data reporting and targeted discovery of data based on RO_2_ fate, which supports development of alternative model frameworks that can capture more diverse reactions pathways in the current and future atmosphere (Figure 6B). These examples represent areas in which ICARUS can grow with the needs of the community.

Data mining and model development activities related to machine learning^62−64^ will benefit from the increasing availability of high-quality data as provided by ICARUS. In particular, machine learning algorithms gain more information each time there is a new “phase change” or time discontinuity with distinct characteristics that can be observed to effect a measurable system impact.^65^ These phase changes occur quite often in each experiment, for example, each time lights are turned on or off, a reactant introduced, or there is a temperature or humidity change that will lead to a measurable impact in the data observations. Thus, datasets from chamber experiments may be excellent tools on which to train machine learning frameworks to simulate complex processes such as secondary organic aerosol formation, gas phase oxidation, or heterogeneous chemistry, as a complement to traditional modeling tools.

## 5 Data Availability

Data described in this work are freely available on the ICARUS website. The reuse of ICARUS shared data in any publication requires notification of the PI and other terms as specified by the Data Use Policy (Section S2). Disseminated reuse of ICARUS data require citation to the data using digital object identifiers (DOIs) of the experiments and to this publication. Data DOIs are assigned by DataCite (https://datacite.org).^66^ All Experiment DOIs associated with a single Experiment Set are shown on the webpage of the Set ID for ease of tracking. The repository DOI (http://doi.org/10.17616/R31NJN8W) is assigned through the re3data registry (https://re3data.org).^67^

## Supporting Information Available

ICARUS user guide; ICARUS data policy; example of experiment timeline; example of the experiment view page on the ICARUS website; and example of the ICARUS data format (PDF)

## Notes

^◆◆^ Now an independent consultant.
