# Supplementary material for: Overview of ICARUS—A Curated, Open Access, Online Repository for Atmospheric Simulation Chamber Data
Source: ACS Earth Space Chem. 2023 May 16;7(6):1235–46. doi: 10.1021/acsearthspacechem.3c00043 (PMC10278178; doi:10.1021/acsearthspacechem.3c00043)
Supplement: Supplementary file 1 — sp3c00043_si_001.pdf [file sp3c00043_si_001.pdf]

Supplemental Information for "Overview of ICARUS – a curated, open access, online repository for atmospheric simulation chamber data"

Tran B. Nguyen,[1*] Kelvin H. Bates,[1,2] Reina S. Buenconsejo,[3] Sophia M. Charan,[3] Eric E. Cavanna,[4§] David R. Cocker III,[5] Douglas A. Day,[6,7] Marla P. DeVault,[6,7ψ] Neil M. Donahue,[8,9,10] Zachary Finewax,[6,7Ω] Luke F. Habib,[9] Anne V. Handschy,[6] Lea Hildebrandt Ruiz,[11] Chung-Yi S. Hou,[12£] Jose L. Jimenez, [6,7] Taekyu Joo,[13Φ] Alexandra L. Klodt,[14] Weimeng Kong, [3ξ] Chen Le,[5] Catherine G. Masoud,[11] Matthew S. Mayernik,[12] Nga L. Ng,[13,15,16] Eric J. Nienhouse,[12] Sergey A. Nizkorodov,[14] John J. Orlando,[17] Jeroen J. Post.,[4] Patrick O. Sturm,[18†] Bridget L. Thrasher,[12] Geoffrey S. Tyndall,[17] John H. Seinfeld,[3,19] Steven J. Worley,[12] Xuan Zhang, [17‡] Paul J. Ziemann[6,7]

1. Department of Environmental Toxicology, University of California Davis, Davis CA 95616
2. Center for the Environment, Harvard University, Cambridge, MA 02138
3. Division of Chemistry and Chemical Engineering, California Institute of Technology, Pasadena CA 91125
4. Information and Educational Technology, University of California Davis, Davis CA 95616
5. Dept. Chemical and Environmental Engineering, University of California Riverside, Riverside, CA 92507
6. Cooperative Institute for Research in Environmental Sciences, University of Colorado Boulder, Boulder, CO 80309
7. Department of Chemistry, University of Colorado Boulder, Boulder, CO 80309
8. Department of Chemistry, Carnegie Mellon University, Pittsburgh, PA 15213
9. Department of Chemical Engineering, Carnegie Mellon University, Pittsburgh, PA 15213
10. Department of Engineering, and Public Policy, Carnegie Mellon University, Pittsburgh, PA 15213
11. McKetta Department of Chemical Engineering, The University of Texas at Austin, Austin, TX 78712
12. Data Stewardship Engineering Team, National Center for Atmospheric Research, Boulder, CO 80307
13. School of Earth and Atmospheric Sciences, Georgia Institute of Technology, Atlanta, GA 30332
14. Department of Chemistry, University of California Irvine, Irvine, CA 92697
15. School of Chemical and Biomolecular Engineering, Georgia Institute of Technology, Atlanta, GA 30332
16. School of Civil and Environmental Engineering, Georgia Institute of Technology, Atlanta, GA 30332
17. Atmospheric Chemistry Observations and Modeling, National Center for Atmospheric Research, Boulder, CO 80305
18. Air Quality Research Center, University of California Davis, Davis CA 95616
19. Division of Engineering and Applied Science, Calif. Institute of Technology, Pasadena, CA 91125

[§] Now an independent consultant
ψ Now at Minnesota Department of Health, 625 Robert St. N. P.O. Box 64975. St. Paul, MN 55164
[Ω] Now at NOAA Chemical Sciences Laboratory, 325 Broadway R/CSL, Boulder, Colorado 80305
[£] Now at Ronin Institute, 127 Haddon Pl, Montclair, New Jersey 07043
[Φ] Now at Yale University, PO Box 208236, New Haven, CT 06520
[ξ] Now at the California Air Resources Board, 1001 I Street, Sacramento, CA 95814
[†] Now at University of Southern California. 3470 Trousdale Parkway, Los Angeles, CA 90089
[‡] Now at UC Merced, 5200 Lake Rd, Merced, CA, USA 95343-5001

*Correspondence to: tbn@ucdavis.edu

Section S1. ICARUS User Guide

S1.A. Accounts

Each Data Contributor group is required to create a free account on the ICARUS website, which is validated using the provided email address, and to log in prior to proceeding with the subsequent steps. A group of researchers may choose to use a single account for the group or to create unlimited individual accounts, which can be linked through the account sharing feature of ICARUS, wherein sharing is achieved through linking account email addresses. One or more accounts may create the data entities for a group, and other accounts may access and edit these entities through the shared access portal. Thus, all members of a research group or organization may contribute data.

S1.A. Organization

First, the Data Contributor team defines an Organization, which is then assigned a unique Organization ID within the repository. Descriptions of the Organization include (with asterisks* denoting required fields here, and throughout):

- Lab name*; lab affiliation*; lab activeness (yes/no)*; principle investigator (PI) name(s)*, contact information*, and ORCID iD;[32] data manager name(s)* and contact information*; and data terms of use* that are defined by each Organization

The Organization may be a lab of a single PI (e.g., “Caltech Atmospheric Chamber” or “Smith Lab”), or a bigger organization within a research institution that comprises the labs of multiple PIs (e.g., “Center for Atmospheric Particle Studies” at Carnegie Mellon, or “Center for Environmental Research and Technology” at UC Riverside). Data access guidance from each Organization helps ensure that the data are used and interpreted accurately, and provides incentive for researchers to contribute to the repository. The Organization may wish to provide their own data access statements, or use the default terms of use defined by ICARUS:

“The Principal Investigator (PI) and institution retain any and all rights to the data that they generated. Use of ICARUS shared data in published materials or materials under review, including peer-reviewed publications or submissions, conference presentations, reports, and proposals, requires notification of the PI. For materials meant for dissemination that significantly depend on the data shared by specific PIs, the database user is required to offer the specific PI(s), and associated coworkers, co-authorship in said publications. When ICARUS data are used in

published materials, the data should be cited and the DOI for the Experiment or Experiment Set should be provided in the Data Availability statement."

S1.B. Instruments

Instruments should be described first, and can then be linked to Chambers. Instruments define an Instrumental Dataset that will be expected or available for upload from experiments performed in the Chamber. For example, if an "Ozone Monitor" Instrument is defined and linked to a Chamber, then any Experiment performed in the Chamber will have a pre-defined space for upload of "Ozone Monitor" data. Instruments are each assigned unique Instrument IDs. Descriptions of an Instrument include:

- Instrument name* (short name and full name); descriptions of what is being measured by the Instrument*; sampling protocols (online/offline)*; manufacturer information*; data recording information (time resolution*, software, averaging, etc.); detection limits*; sensitivities to temperature and humidity; tubing characteristics (length, inner diameter, and material); flow rates; chemical identification methods; chemical quantification methods (including calibration methods); data analysis protocols; calibration schedule and drift; measurement uncertainties; uncertainty estimation methods*; known interferences; links to supplemental information

In practice, Instruments that provide data may be shared between different chambers and/or flow reactors within a research lab depending on the active projects. Bigger organized research units may share Instruments between labs, especially if the Instrument is not owned by any particular lab PI. Thus, the associations between Instruments and Chambers in ICARUS are flexible.

S1.C. Chambers

The entity termed Chamber describes both the reactor and the entire physical enclosure infrastructure and associated items needed to conduct chamber research (e.g., lights, temperature/humidity control, air filtration/flow, sampling apparatus, etc.). If the chamber is replaceable, e.g., inflatable Teflon chambers, a new entry is not required for each replacement. The physical characteristics of each Chamber are described in the intake form, and each entry is assigned a Chamber ID. Descriptions of a Chamber include:

- Chamber name*; Chamber characteristics (shape, volume*, area*, flow mode*, material*, etc.); replacement schedules; cleaning frequency* and methods; chemical and particle backgrounds; mixing* and filtration methods; temperature control* and monitoring*; humidity

control* and monitoring; ability to conduct particle experiments*; lighting characteristics*; and linkages to Instruments that exist in the Organization

When creating a Chamber, a listing of previously-described Instruments is automatically populated, and linked by marking a checkbox. Thus, any Instrument only needs to be described once, even if it is used for multiple Chambers. After linking an Instrument to a Chamber, any Experiment performed within that Chamber will allow for the upload of an associated dataset file (restricted to .csv) for the linked Instrument. For example, if an Instrument called "Ozone Monitor" was linked to the Chamber, then space for the Ozone Monitor dataset is automatically available for upload when creating an Experiment within that Chamber. However, depending on whether that Instrument was sampling during the Experiment, the Data Contributor may choose whether or not to upload that dataset. Leaving the upload space empty is an option if there is already at least one other Dataset uploaded for the Experiment to satisfy the minimum requirement. Multiple Datasets are allowable for each linked Instrument, and more Datasets may be easily added.

S1.D. Experiment Sets

Experiment Sets group individual Experiments using any logic defined by the Data Contributor. These entities are tracked by a Set ID. There are different granularities at the Experiment Set level that may exist for each Organization, which provides flexibility for how Organizations prefer to manage data. The following are some examples of how various experimental data can be grouped in Experiment Sets, depending on the needs or preferences of the Organization.

- Grouping based on Reaction Type – every experiment that studies a particular chemical or physical reaction may be grouped together, e.g., all Ozonolysis experiments, or all Vapor Partitioning reactions.
- Grouping based on Publication – all reactions performed for a particular publication may be grouped together. If done this way, any Publications that are linked to the Experiment Set can be more clearly associated with the data.
- Grouping based on Researcher – all reactions performed by a particular lab member may be grouped together. This may be beneficial for legacy data from past students/postdocs/staff, or for organizations that prefer to maintain their records in relation to people instead of projects.
- Grouping based on Grants or Projects – all data that were obtained while supported by a certain funding source may be organized together for ease of reporting.
- Grouping based on Collaborative Study – data obtained in the chamber as part of a themed study by researchers that are both internal and external to the Organization can be grouped

together. For example, the 30-day FIXCIT[24] chamber studies in 2014 that brought several field instruments to the Caltech chambers[18] to study biogenic oxidation chemistry are grouped together in one Set.[33]

Description of an Experiment Set includes:

- Experiment Set name*; Experiment Set description*; time formats and units used in this Set; collaborator names and affiliations; and associated Publication.

Numerous external collaborators on a project can be listed on an optional basis. All Publications (Section 3.1.2.f) defined in the system are populated at the Experiment Set page; the Data Contributor places a check next to the Publications associated with the Set.

S1.E. Experiments

Each Experiment in an Experiment Set is the individual records of the chamber investigations. Each experimental record, which may contain numerous datasets, will be assigned a unique Experiment ID.

Description of an Experiment includes:

- **General Fields:** Experiment category(s)*; reaction type(s)*; experimental start date (calendar entry)*; temperature*; relative humidity*; pressure.
- **Seeding and Reaction:** Seeded experiment (yes/no)*; type of seed; initial seed concentration value; seed concentration units (μg/m^3 or particles/m^3); Fuchs adjusted surface area;[34] condensation sink rate coefficient; oxidant name*; initial oxidant value*; oxidant concentration unit (μg/m^3, molec/cm^3, ppt, ppb, ppm)*; reactant name(s); reactant functional groups; reactant concentration; reactant concentration units (μg/m^3, molec/cm^3, ppt, ppb, ppm); peroxy radical (RO_2) main fate*;[35] RO_2 lifetime (s).
- **Description and Timeline:** Experiment title extension (Section 2.1.3); Experiment goals*; Experiment summary*; additional notes; Chamber characterizations (Section 2.1.2.g); Publications (Section 2.1.2.f); Experiment timeline file upload*; make my data public* (yes/no, and embargo end date).
- **Datasets:** Dataset upload spaces are available for each linked Instrument, and at least one uploaded dataset is required for each Experiment. Multiple datasets per instrument may be accommodated by the "Add dataset" function.

For the longer text entry fields such as "Experiment goals", a minimum number of words is required to promote descriptiveness. Any Characterization files (Section 3.1.2.g) that have been uploaded for the Chamber are available to attach to the experiment using drop down menus.

The main requirements for uploads of Experiments are the Instrumental Dataset files and Timeline file. The **Instrumental Dataset** files are data files in comma separated values (.csv) format that have been processed to their final ready-to-use forms (e.g., raw signals that have been baseline corrected, calibrated and converted to concentration units). Some instruments will export raw data that are ready to use, as the required processing had been done during the data collection process with the data collection software, and some raw data will require post processing by the Data Contributor prior to their publication in ICARUS and/or a peer-reviewed manuscript. The processed data can either be directly exported in .csv format, or converted using a programmatic script. Non-numeric headings can be accepted; and there is an option to indicate the number of heading rows that should be expected for each type of Instrumental Dataset when defining the Instrument entity; this is so data reader scripts can import non-numeric headers using different code.

The **Timeline** lists chronological experimental actions that occurred during the Experiment, so that the data taken by the Data Contributor group can be appropriately interpreted by the Data User. The upload of the Timeline requires a two column .csv file, and once the file is uploaded, a quality check is formed to verify the presence of exactly two columns upon file upload to ensure that erroneous commas are not present in the file. The first column's expression of time should match how time is reported in the Instrumental Datasets. Actual Timelines may be more or less detailed than the examples given here (**Table S1**); however, any injections of reactants, sampling actions (start, stop, interruptions), changes in irradiation or environmental conditions, or any action by the experimentalists that are observable in the data should be described in the Timeline.

The input of **Reactant Name(s)** is controlled through usage of the PubChem Database. All user-inputted synonyms are linked through the PubChem ID (PID) and will automatically retrieve the "preferred" or "common name" synonym as defined by PubChem. For example, when the Data Contributor enters a reactant name such as "2-pinene", ICARUS uses an Application Programming Interface (API) to validate this reactant name against PubChem and retrieve its corresponding PID (which, this example, is 6654). This PID retrieval will return the common name "alpha-pinene," which updates the user entry as the displayed value in the Reactant Name field. Mixtures of compounds that represent commercially available mixtures (PubChem Substances) such as "Diesel

Fuel #2" are also acceptable, or can be defined as multiple pure Compounds if the mixture composition is known by the experimentalist. If the reactant mixture is unknown (e.g. hydrocarbon emissions from real trees, or fire emissions), the Data Contributor may use the custom entry form to define their own reactant(s). In each Experiment, a multitude of Reactants may be entered. A valid entry for a reactant is the term "Aerosol," which is a particle-phase chemical or chemical mixture. Aerosols may also be defined through the "Seed" particle field through a drop-down menu of controlled vocabulary.

S1.F. Publications

Works in the scientific literature that are associated with the uploaded data are entered into the system as Publications, which are tracked by a Publication ID. A full list of all publications associated with ICARUS data are found under the "References" button on the top toolbar of the website. Publications can be linked at either the Experiment Set level or the Experiment level. This is most useful for Experiment Sets that are organized by published works in the literature; a linkage to a Publication at the Experiment Set level is automatically propagated to the level of Experiments unless otherwise noted. It is most useful to upload data to ICARUS prior to manuscript submission to include the data DOIs in Data Availability statements, and link the Publication DOI to the Experiment Set or Experiment(s) after the manuscript has been accepted.

Each Publication requires the following input:

- Title*, author list*, publication DOI*, abstract, journal short name*, publication year*, publisher*, and miscellaneous notes

The manual entry of publication details is necessary at this time due to inconsistencies in publication metadata retrieved from APIs. API retrievals using publication digital object identifiers (DOI) resulted in significant variation in JavaScript Object Notation metadata fields from journal to journal, which appear to depend upon the journal's country of origin and publisher (e.g., Resume vs. Abstract vs. Summary). This underscores the need for global standards for scientific publication metadata.

S1.G. Characterizations

Experiments performed to characterize the physical constants associated with the Chamber are called Characterizations. The ICARUS website provides documentation on chamber

characterization protocols that were developed collaboratively by the pilot cohort of Data Contributors. These protocols may be used as guides for future researchers and as references for general methods. ICARUS currently allows three types of Characterizations: (1) Light Flux characterizations are quantifications of the emission photon flux from the lamps surrounding the Chamber; (2) Particle Wall Loss characterizations are quantification of loss rates of particles to the chamber walls, which may depend on the particles' diameter and composition as well as chamber conditions such as temperature, relative humidity, light exposure, and other factors; (3) Vapor Wall Loss characterizations are quantifications of loss rates of organic and/or inorganic gases to the chamber walls, which depend on the chemical structure of the volatile compound, the physical characteristics of the chamber, and environmental conditions of the chamber. Descriptions of each Characterization dataset include:

- <u>Light Flux Characterization</u>: Name of characterization experiment*, date of characterization experiment* (calendar entry), Instrument used to perform characterization, months since instrument calibration, measurement technique description, photolysis frequency of NO_2 loss, photolysis frequency of H_2O_2 loss, photolysis frequency of O_3 loss, total number of lights used in the characterization, percentage of lights on*, light intensity, light source type(s), position and pattern of lights around the chamber*, data collection description*, data analysis description*, link to supplemental information, additional notes, and Characterization file upload*

- <u>Particle Loss Characterization:</u> Name of characterization experiment*, date of characterization experiment* (calendar entry), Instrument used to perform characterization, months since instrument calibration, temperature*, relative humidity percentage*, particle composition* (controlled), particle measurement size range*, particle generation method*, wall loss experiment description*, wall loss rates calculation description*, frequency of particle loss experiments, wall loss correction description*, link to supplemental information, additional notes, and Characterization file upload*

- <u>Vapor Loss Characterization:</u> Name of characterization experiment*, date of characterization experiment* (calendar entry), Instrument used to perform characterization, months since instrument calibration, temperature*, relative humidity percentage*, volatile compound name or class*, volatile compound source* (controlled), wall loss experiment description*, vapor loss calculation method*, parametrization (yes/no)* and description, link to supplemental information, additional notes, and Characterization file upload*

Generally one set of Characterizations will serve multiple Experiment Sets or Experiments (e.g., one light flux characterization may be used throughout all experiments). Characterizations can be linked at the Experiment Set level and at the Experiment Level. If Characterizations are linked at the Experiment Set level, the linkages are propagated to all of the Experiments. Alternatively, some experiments in a Set may associate with different (or multiple) Characterizations. It is common, for example, for Experiments that vary in temperature or relative humidity to require a different file for Particle Wall loss characterizations. In this case, overrides are available at the Experiment Level by selecting different Characterizations from a drop-down menu in the Experiment data entry form.

S1.H. Task Automation

This section describes the Clone Tool, Move Tool, Autogenerated Experiment Names, and Draft Mode. Cloning is available for multiple entities: Chambers, Instruments, Experiment Sets, Experiments, and Characterizations. In order to Clone an entity, click on the cloning icon (copy icon) in Experiment View tables, or click the "Actions" button and select "Clone." Cloning which will bring up a template of the entity with the majority of the information intact. The information will need to be updated for the new entity. The cloning feature does not accept duplicates in general, and will return an error if nothing is changed throughout the cloning process. Cloning an entity creates a new ID in the system. Cloning an Experiment, for example will remove the date entry, which will need to be entered again, to avoid duplications in this field. Cloning an experiment will also change the title, adding the term "clone" in order to prompt the user to update the title accordingly. Cloning an entity will create a new entity ID within the ICARUS system.

The Move tool is available for each Experiment. Under the "Actions" available for Experiments, select "Move." This will associate the Experiment with a new Experiment Set that will be selected by the Data Contributor. This is different than Clone because it does not create a new Experiment ID; the existing Experiment ID will now associate with a different Experiment Set ID.

The core structure for Experiment names is auto-generated with the general syntax: ICARUS_Organization_Exp.Date_Reactant(s)_Oxidant_Seed." The entries to each name field are used, separated by a slash in the event of multiple entries such as multiple reactants. Null responses are substituted by terms such as "NoSeed" or "NoOxidant" that is affixed to the name. The automatic generation of names is done to improve both machine and human readability while reducing the workload on the user. The Data User has enough information in the title to distinguish

downloaded datasets by eye. The experiment title may be extended with an optional Experiment Title Extension freeform text field. For example, if the Data Contributor group performed experiments with identical reactants, oxidants, seed particles but with different relative humidity, then they may opt to add "RH55" or "RH80" to the title to differentiate the Experiment. These title extensions are aimed to increase readability to human eyes and facilitate the search and discovery process.

The process of saving data entry and upload progress for future work is achieved by a "save as draft" feature on the Create Experiment task. Drafts are not published and ignore errors of incomplete fields. The Data Contributors can come back to the draft to continue their work at any time, and save the draft again, or publish the Experiment.

S1.I. Data Format

ICARUS data files are written in the CSVY (csv-yaml) file format,[44] which combines a nested data serialization language called YAML[45] with comma-separated values. The YAML portion of the file (called the YAML front matter) is used to display metadata from the website, and the CSV portion of the file is used to display processed data uploaded by the Data Contributor. The file extension .txt is used to be more easily recognizable by most computers and easily viewable with many software programs. An example of the CSVY data format is shown in **Figure S2**. A downloaded data packet (compressed .zip file) for one single Experiment contains:

- A folder called "Characterization" that includes a YAML-based .txt file for each available Characterization dataset linked to the Experiment
- A folder called "Publications" that includes a YAML-based .txt file for each available Publication linked to the Experiment (there is no CSV portion to this file)
- A folder called "Datasets" that includes a YAML-based .txt file for each associated Instrumental Dataset uploaded to the Experiment, where the YAML front matter describes the metadata details that were entered into the website under Instrument.
- An Experimental Metadata YAML-based .txt file (whose name always starts with "Experiment_") where the YAML front matter describes the experimental details that were entered into the website under Experiment and the CSV portion shows the Experimental Timeline.
- A YAML file called "manifest.txt" that lists the download timestamp, Experiment ID, all contents of the download and a permalink to the Experiment on ICARUS.

Multiple unrelated Experiments can be queued for download by adding to a "My Downloads" list, which will reorganize all downloaded files by Organization, and compress the data packages into a single .zip file.

S1.J. Downloading Data

Data Users may choose to download experiments based on the results of the search and discovery table, or view individual experiments first before downloading (Section 3.2.1). For the process of downloading data directly from the results table (**Fig. 3**), two Actions are listed with every Experiment: (1) "Download this Experiment" which gives the Data User an estimation of the size of the download in megabytes (MB) and proceeds to download the single experiment immediately, or (2) "Add to My Downloads" which queues the Experimental for a batch download together with other selected Experiments. An alternative way to perform multiple downloads is to use the check mark buttons on the search and discovery table, where users can quickly select any and all Experiments that fit their search criteria for a batch download.

Data Users are not required to have an account to peruse data, but will need to undergo a quick and free registration process to download data. The Data User is required to register a valid email address, name, affiliation, and download purpose. The user is also required to check that they have read and understood the Data Use Policy of ICARUS (**Section S2**). The user registration is validated through a link sent to the email address, after which the user can download unlimited datasets. Download statistics are tracked anonymously to quantify data usage rates.

Section S2. The data policy of ICARUS

"*The Principal Investigator (PI) and institution retain any and all rights to the data that they generated. Use of ICARUS shared data in published materials or materials under review, including peer-reviewed publications or submissions, conference presentations, reports, and proposals, requires notification of the PI.*

For materials meant for dissemination that heavily depend on the data shared by specific PIs, the data user is required to offer the specific PI(s), and associated coworkers, co-authorship in said publications.

When ICARUS data are used in published materials in any capacity, ICARUS should be acknowledged as the source of the data with the following statement "Data used in this work were in part sourced from the ICARUS database that was developed with support by the National Science Foundation AGS program (https://icarus.ucdavis.edu)." A citation to the ICARUS paper is encouraged. Furthermore, all data used in the manuscript must be cited using the Digital Object Identifiers (DOIs) of the Experiment or the Experiment Set."

Table S1: Example of an experimental timeline for the hypothetical isoprene photoxidation experiment described in Methods.

Time (local hh:mm)	Action (no commas)
9:00	Humidify chamber
9:05	Turn on all instruments
11:00	Chamber is at 50% relative humidity. Stop humidification. Cap chamber and start injection of 50 uL hydrogen peroxide in water (50 wt%) for 30 minutes
11:05	Inject 5 uL of isoprene (99%) with 5 slm zero air for 15 minutes
11:20	Mixing air in for 10 minutes
11:30	Turn off all injection flows
11:35	Lights on (100%)
13:18	Sampling from instrument X is interrupted by a sample inlet clog
13:32	Sampling resumes again for instrument X
15:30	Lights off. Flush chamber and shut down instruments.

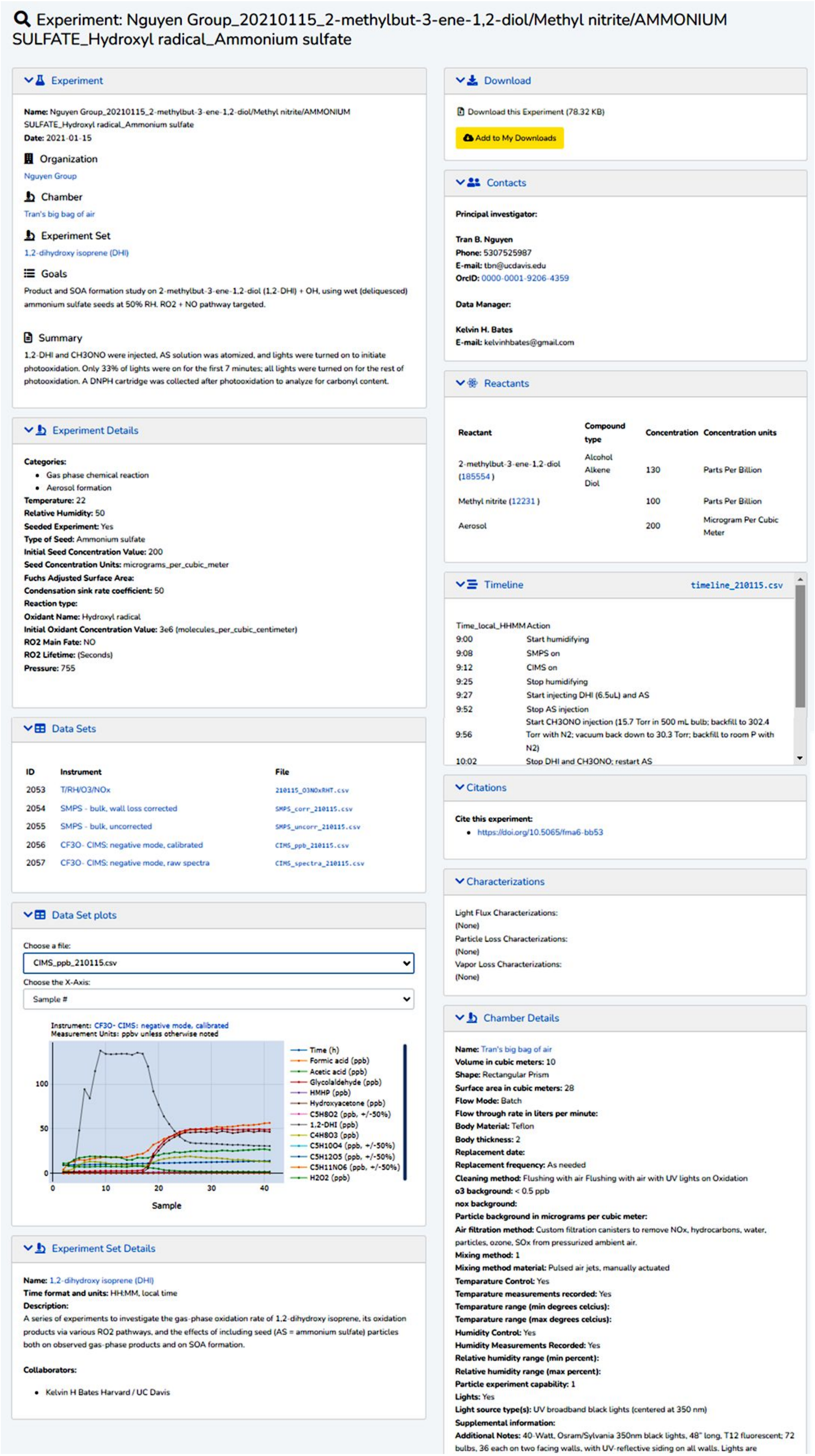

Figure S1: Example of an Experiment View page on the ICARUS website.

```
---
instrument_id: '480'
data_file_original_name: '211116_CIMS_ppb.csv'
skip_headers: '1'
charts: ''
headers:
    - 'Time (h)'
    - 'Formic acid (ppb)'
    - 'Acetic acid (ppb)'
    - 'Glycolaldehyde (ppb)'
    - 'Hydroxyacetone (ppb)'
    - 'isoprene diol (ppb)'
dataset_id: '3750'
experiment_link: 'https://icarus.ucdavis.edu/chambers/552/experiments/691'
experiment_set_link: 'https://icarus.ucdavis.edu/chambers/552/experimentSets/226'
chamber_link: 'https://icarus.ucdavis.edu/chambers/552'
dataset_created_at: '2021-12-08 09:49:43'
dataset_updated_at: '2021-12-08 09:49:43'
instrument_link: 'https://icarus.ucdavis.edu/chambers/552/instruments/480'
instrument:
    id: '480'
    short_name: 'CF3O- CIMS: negative mode, calibrated'
    full_name: 'chemical ionization mass spectrometer w/ CF3O- reagent ion'
    manufacturer: 'Varian'
    model: '1200'
    year: ''
    data_recording_software: 'Varian workstation'
    data_analysis_software: 'Matlab'
    raw_data_time_resolution: '0.125 s'
    analysis_data_averaging: '0.5 s averages of raw data'
    detection_limit: '100 ppt'
    quantification_limit: ''
    sensitivity_to_temperature: 'minor'
    sensitivity_to_relative_humidity: 'ions are independently calibrated for RH'
    sampling_method: 'Direct sampling'
    sample_preparation_method: ''
    sample_residence_time_in_seconds: ''
    length_of_tubing_in_cm: '100'
    flow_rate: '2 lpm'
    tubing_material: 'FEP Teflon'
    chemical_identification_method: 'ion fragmentation in triple quadrupole'
    data_analysis_method: ''
    quantification_method: 'Baseline correction, dilution correction, humidity-dependent calibration factor'
    calibration_method: 'Compound-dependent: permeation tube, commercial or synthetic standards'
    calibration_drift_estimate: 'Low (on the order of weeks)'
    calibration_schedule: 'As Needed'
    uncertainty_estimation_method: 'Uncertainties are estimated from propagating uncertainties in the steps of the absolute calibrations'
    known_interferences: ''
    link_to_supplemental_information: 'St. Clair et al. (DOI 10.1063/1.3480552)'
    additional_notes: ''
    organization_id: '559'
    created_at: '2021-08-09T20:40:57.000000Z'
    updated_at: '2021-08-10T01:34:00.000000Z'
    column_descriptors: ''
    sampling_protocol: 'Online'
    skip_headers: '1'
    tubing_inner_diameter: '0.16 in'
    what_is_measured: 'oxidized VOCs (especially alcohols, hydroperoxides, acids, nitrates)'
    measurement_uncertainty: '+/- 30% unless otherwise noted'
    measurement_units: 'ppbv unless otherwise noted'

---
Time (h),Formic acid (ppb),Acetic acid (ppb),Glycolaldehyde (ppb),Hydroxyacetone (ppb),isoprene diol (ppb)
9.712696101,2.404409273,166.5224292,1.680980908,0.985231525,0.613423512
9.823392858,11.91878842,20.39602418,1.498654371,0.846645755,21.61916523
9.936939293,13.15791267,16.64344002,1.599010258,0.848786872,49.4480113
10.04814512,14.5643267,14.5825955,1.651717957,0.861010014,127.3518142
10.16172175,14.74803725,12.79875147,1.713184027,0.837882113,178.6027867
10.27614015,16.64918336,13.52809396,1.901251218,0.944484147,270.2609196
[illegible]
```


Figure S2. Data Format example for an Instrumental Dataset. The full data in the CSV part of the file is cut off for brevity.
